# P-263. Sodium dichloroisocyanurate: A promising candidate for the disinfection of resilient drain biofilm

**DOI:** 10.1093/ofid/ofae631.467

**Published:** 2025-01-29

**Authors:** Abbbie Martin, Rodney E Rohde, Tom O’Mahony, Natasha Doyle

**Affiliations:** Kersia Ireland, Wexford, Wexford, Ireland; Texas State University, San Marcos, Texas; Kersia Ireland, Wexford, Wexford, Ireland; Kersia Ireland, Wexford, Wexford, Ireland

## Abstract

**Background:**

Biofilms are complex multicellular communities of microorganisms which can lead to a variety of human diseases and infections. Biofilms consist of microbes which are contained within a protective extra polymeric substance layer, resulting in increased resistance to disinfectants. From an economic perspective, it is estimated that potentially deadly biofilms, which cause 1.7 million hospital acquired infections (HAIs) each year in the US, can result in costs of $11.5 billion in treatment. Biofilms thrive in a variety of diverse environments, including hospital sinks and drains, which can pose a significant threat to patient safety. Sink drains maintain one of the highest burdens of antibiotic resistant bacteria amongst all other surfaces, with *Pseudomonas aeruginosa* consistently being the most prominent species found within ICU sink drains. Studies have shown that by completely removing sinks from patient rooms, there is a significant reduction in the colonisation of gram-negative bacteria, a leading cause of HAIs.

**Methods:**

The aim of this study was to determine if three hospital-grade disinfectants were capable of killing biofilm, and as such, if they could be used as potential drain disinfectants. Disinfectants were comprised of Sodium Dichloroisocyanurate (NaDCC), Peracetic acid and bleach-based disinfectants. *Pseudomonas aeruginosa* biofilms were cultivated using the CDC biofilm reactor, a standardised method for determining biofilm efficacy within the United States.

**Results:**

The NaDCC product was successful in completely killing the biofilm (using a one-minute contact time), resulting in a total 8.70 log reduction. Peracetic acid reduced biofilm by 3.82 logs, followed by bleach, which had the lowest reduction at 3.78 logs.

**Conclusion:**

Drain disinfection is critical to ensure patient safety and reduce the instances of HAIs. This experiment can help in predicting the effectiveness of three hospital disinfectants for drain applications. NaDCC could provide a reliable solution for drain applications and subsequent patient safety.

**Disclosures:**

**Abbbie Martin, BsC**, Kersia Ireland: Employee **Tom O'Mahony, PhD**, Kersia Ireland: Employee **Natasha Doyle, PhD**, Kersia Ireland: Employee

